# Association of sleep among 30 antidepressants: a population-wide adverse drug reaction study, 2004–2019

**DOI:** 10.7717/peerj.8748

**Published:** 2020-03-11

**Authors:** Andy R. Eugene

**Affiliations:** 1 Independent Researcher, Kansas, United States of America; 2 Independent Neurophysiology Unit, Department of Psychiatry, Medical University of Lublin, Lublin, Poland

**Keywords:** Sleep, Somnolence, CYP2C19 poor metabolizers, CYP2D6 poor metabolizers, Glymphatic system, Insomnia, Clinical pharmacology, Depression and anxiety, Pharmacogenomics, Psychopharmacology

## Abstract

**Background:**

Sleep is one of the most essential processes required to maintain a healthy human life, and patients experiencing psychiatric illness often experience an inability to sleep. The aim of this study is to test the hypothesis that antidepressant compounds with strong binding affinities for the serotonin 5-HT2C receptor, histamine H1 receptors, or norepinephrine transporter (NET) will be associated with the highest odds of somnolence.

**Methods:**

Post-marketing cases of patient adverse drug reactions were obtained from the United States Food and Drug Administration Adverse Events Reporting System (FAERS) during the reporting window of January 2004 to September 2019. Disproportionality analyses of antidepressants reporting somnolence were calculated using the case/non-case method. The reporting odds-ratios (ROR) and corresponding 95% confidence interval (95% CI) were computed and all computations and graphing conducted in R.

**Results:**

There were a total of 69,196 reported cases of somnolence out of a total of 7,366,864 cases reported from January 2004 to September 2019. Among the 30 antidepressants assessed, amoxapine (*n* = 16) reporting odds-ratio (ROR) = 7.1 (95% confidence interval [CI] [4.3–11.7]), atomoxetine (*n* = 1,079) ROR = 6.6 (95% CI [6.2–7.1]), a compound generally approved for attention deficit hyperactivity disorder (ADHD), and maprotiline (*n* = 18) ROR = 6.3 (95% CI, 3.9–10.1) were the top three compounds ranked with the highest reporting odds of somnolence. In contrast, vortioxetine (*n* = 52) ROR = 1.3 (95% CI [1.0–1.8]), milnacipran (*n* = 58) ROR = 2.1 (95% CI [1.7–2.8]), and bupropion (*n* = 1,048) ROR = 2.2 (95% CI [2.1–2.4]) are least significantly associated with somnolence. Moreover, levomilnacipran (*n* = 1) ROR = 0.4 (95% CI [0.1–2.9]) was not associated with somnolence.

**Conclusion:**

Among the thirty tested antidepressants, consistent with the original hypothesis, amoxepine has strongest 5-HT2C receptor binding affinity and has the highest reporting odds of somnolence. Atomoxetine, ranked second in reporting odds of somnolence overall, binds to the NET with with the strongest binding affinity among the thirty compounds. Mirtazapine, a tetracyclic antidepressant, was ranked 11th in reporting odds of somnolence and had the strongest H1 receptor binding affinity. This study provides an informative ranking of somnolence among thirty antidepressant compounds with an already wide array of clinical indications as well as provides insight into potential drug repurposing in psychopharmacology.

## Introduction

The inability to sleep, or insomnia, has been historically reported in patients experiencing depression, anxiety, alcoholism, suicidal ideation, suicide attempts, and substance abuse (Roane & Taylor, 2008; Brower, 2003; Chakravorty et al., 2014; Don Richardson et al., 2018; Taylor et al., 2005; Yaya et al., 2018). In fact, sleeping disturbances may be so pervasive in chronic cases of the aforementioned patient groups, that the most recently approved antidepressant compound, indicated for treatment-resistant depression and intended to be used in conjunction with an oral antidepressant, is the S-enantiomer of ketamine, a racemic compound of R- and S-ketamine which is used in anesthesia (DailyMed., 2019b; White et al., 1985). Similarly, another effective compound used in treating patients with treatment-resistant depression and depressive episodes associated with Bipolar I Disorder, combines olanzapine, an antipsychotic that shows gender differences when binding to the dopamine D2, with fluoxetine and recommended to be administered nightly due to olanzapine’s impact on sleep (DailyMed., 2019a; Eugene & Masiak, 2017; Lazowski et al., 2014).

Sleep is reported to be neuroprotective by clearing out metabolic waste products via the brain’s glymphatic system and a recent systematic review and meta-analysis found that exercise improves the quality of sleep (Banno et al., 2018; Eugene & Masiak, 2015; Xie et al., 2013). Antidepressants are prescribed for an array of clinical indications and differ in pharmacokinetics and pharmacodynamics (Eugene, 2019; Wong et al., 2016). Irrespective of the clinical diagnoses for which an antidepressant compound is indicated, as a result of modulation of monoamine transporters (e.g., norepephrine (NET), serotonin (SERT), and dopamine (DAT)), binding at various receptor affinities (Ki), altering gene expression, as well as other factors, certain adverse drug reactions are categorically common amongst antidepressants (Eugene, 2019; Eugene, Masiak & Eugene, 2018; Owens et al., 1997; Qin et al., 2019). The term somnolence derives from French and Latin meaning to *sleep*. In patients who are prescribed antidepressants and operate heavy machinery, work in aviation, or require alertness in their profession, excessive somnolence may result in severe adverse outcomes. Similarly, in patients who suffer from major depressive disorder and experience comorbid symptoms of insomnia, anxiety, intrusive and obsessive thoughts, compulsive behaviors, or agitation, antidepressants with off-target pharmacodynamic effects that induce somnolence may be beneficial. Thus, a comparative analysis ranking antidepressants based on the highest and lowest odds of somnolence is an unmet clinical need and informative.

Sleep is one of the most essential processes required to maintain a healthy human life (Cirelli, 2006; Hobson, 2005). Various receptors are implicated when considering effects on somnolence. Compounds blocking alpha-1 adrenergic receptor, histaminergic receptors (H1), muscarinic cholinergic receptors, N-methyl-D-aspartate (NMDA) receptors thereby decreasing glutamate, and also compounds blocking melatoninergic receptors leading to an increase in *γ*-aminobutyric acid (GABA) concentrations, are all well-established to promote sleep (Watson, Baghdoyan & Lydic, 2010; Watson, Baghdoyan & Lydic, 2012). Further, slow wave sleep is found to be predominately mediated by the serotonin 2C (5-HT2C) receptors that are found in the choroid plexus and is the same neuroanatomical location of the alpha-1 adrenergic receptors that promote clearance of metabolic waste from the brain (Sharpley et al., 1994; Szot, 2016). Among various cellular and physiological processes occurring during sleep, a key component is the activation of the brain’s neuroprotective glymphatic system (Jessen et al., 2015). The catecholamine neurotransmitter norepinephrine is established as the primary neurotransmitter that suppresses the brain’s glymphatic system when awake by inhibiting the choroid plexus cerebral spinal fluid flow (Jessen et al., 2015). Psychotropic medications with relatively potent alpha-1 adrenergic receptors binding affinities, selectively inhibit the norepinephrine reuptake transporters, or have a strong affinity for the 5-HT2C receptors in the choroid plexus all have the potential to influence the clearance of the brain’s metabolic waste products improving cognition and sleep (Szot, 2016; Xie et al., 2013).

With this information as a background, the aim of this study is to test the hypothesis that antidepressant compounds with low Ki values—that is strong receptor binding affinities—for the serotonin 5-HT2C receptor, histamine (H1) receptors, or norepinephrine transporter (NET) will be associated with the highest odds of somnolence. To test this hypothesis, I will use post-marketing pharmacovigilance cases reported to the Food and Drug Administration MedWatch program and conduct disproportionality analysis.

## Materials & Methods

Adverse drug event cases were obtained from post-marketing adverse drug reactions reported to the MedWatch program enacted by the United States Food and Drug Administration’s Adverse Event Reporting System (FAERS) (United States Food and Drug Administration, 2018). Institutional Review Board approval was not required due to patient cases being de-identified and available from the FDA website (https://www.fda.gov). Patient cases included all age groups from FAERS reports originating from the first quarter of 2004 and ending in the third quarter of 2019. The primary outcome variable is the reporting odds-ratios of the following thirty antidepressant compounds: amitriptyline, amoxepine, atomoxetine, bupropion, citalopram, clomipramine, desvenlafaxine, doxepin, duloxetine, escitalopram, esketamine, fluoxetine, fluvoxamine, imipramine, levomilnacipran, maprotiline, mianserin, milnacipran, mirtazapine, nortriptyline, olanzapine/fluoxetine, paroxetine, phenelzine, selegiline, sertraline, tanylcypromine, trazodone, venlafaxine, vilazodone, and vortioxetine. Somnolence is the coded preferred term referenced from the Medical Dictionary for Regulatory Activities (MedDRA) for analysis in this study (Brown, Wood & Wood, 1999). In a secondary analysis, the thirty antidepressant compounds are grouped based on the following pharmacological drug class and assessed for reporting odds of somnolence: atypical antipsychotic/selective serotonin reuptake inhibitor, monoamine oxidase inhibitors, non-competitive N-methyl-D-aspartate receptor antagonist, norepinephrine reuptake inhibitor, norepinephrine-dopamine reuptake inhibitor, selective serotonin reuptake inhibitors, serotonin antagonist and reuptake inhibitor, serotonin modulator and stimulators, serotonin-norepinephrine reuptake inhibitors, tetracyclic antidepressants, and tricyclic antidepressants.

### Drug-gene associations

Human phase I and phase II drug metabolizing enzyme data that includes cytochrome P450 (CYP) inhibitor or inducer information are referenced from the Consensus Guidelines for Therapeutic Drug Monitoring in Neuropsychopharmacology Update 2017 and the FDA Drug Development and Drug Interactions Table of Substrates, Inhibitors and Inducers (CDER, 2017; Hefner, 2018).

### Statistical analysis

Disproportionality analyses of psychotropic adverse events resulting in somnolence were calculated using Fisher’s exact test, via a two-by-two contingency table, using the case/non-case method (Egberts, Meyboom & Van Puijenbroek, 2002; Rochoy et al., 2018). A minimum of 1 case is required and the final estimated reporting odds-ratios (ROR) and corresponding 95% confidence interval (95% CI) are reported in the text. All graphical illustrations as well as all computations were performed using R (version 3.5.2, R Foundation for Statistical Computing, Vienna, Austria) (Böhm et al., 2016; R Core Team, 2013). The reporting odds-ratio-95% confidence intervals not crossing a value of one is interpreted as statistically significant.

## Results

From January 2004 to September 2019 there were a total of 7,366,864 cases reported to the FDA Adverse Events Reporting System with 69,196 cases specifically reported as somnolence. Duloxetine (*n* = 1,710) ROR = 3.0 (95% CI [2.8–3.1]) was the antidepressant with the most reported cases of somnolence, while levomilnacipran (*n* = 1) ROR = 0.4 (95% CI [0.1–2.9]) had only one reported case of somnolence and not significantly associated with the adverse event. Amoxapine (*n* = 16) ROR = 7.1 (4.3–11.7), a metabolite of the antipsychotic loxapine, had the highest reporting odds of somnolence among the study compounds. Whereas, atomoxetine (*n* = 1,079) ROR = 6.6 (95% CI [6.2–7.1]), a norepinephrine reuptake inhibitor and primarily approved for attention deficit hyperactivity disorder (ADHD), was found to have the second strongest association with somnolence. Vortioxetine (*n* = 50) ROR = 1.3 (95% CI [1.0–1.8]) was least statistically significantly associated with somnolence among thirty testing antidepressant compounds.

**Figure 1:**
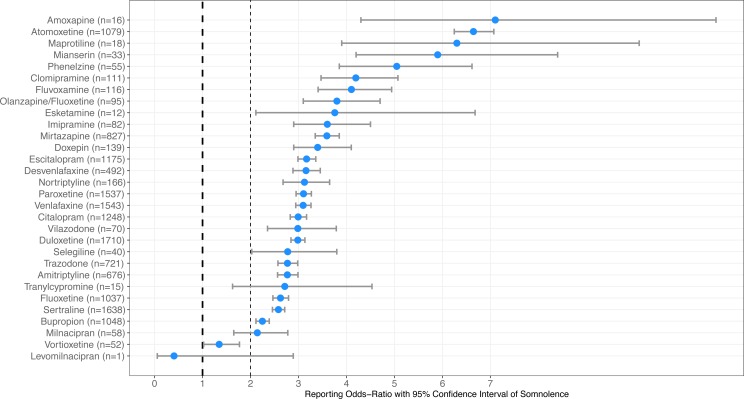
Association of somnolence among 30 antidepressants compounds. Forest plots illustrating reporting odds-ratios (ROR) and 95% confidence intervals of antidepressants ranked from the highest to lowest reporting odds of somnolence reported to the Food and Drug Administration Adverse Events Reporting System during January 2004 and September 2019.

Two marketed antidepressant compounds mainly in treatment-resistant depression were found to have an equal reporting odds-ratio of somnolence: olanzapine/fluoxetine (*n* = 95) ROR = 3.8 (95% CI [3.1–4.7]) and esketamine (*n* = 12) ROR = 3.8 (95% CI [2.1–6.7]). Several compounds, seven groups totaling eighteen antidepressants, were found to have equal reporting odds of somnolence and the results are available in Table 1, sorted from highest to lowest reporting odds of somnolence. Figure 1 illustrates the forest plots of the thirty antidepressants compounds ranked based on reporting odds of somnolence to the FAERS.

**Table 1:** Reporting Odds-Ratios (ROR) and 95% confidence intervals of 30 antidepressant compounds included in the study. There were a total of 69,196 reports of somnolence and 7,366,864 total cases reported overall from January 2004 to September 30, 2019 to the Food and Drug Administration Adverse Events Reporting System.

Antidepressant	**Somnolence cases**	**Other reported cases**	**Somnolence ROR (95% CI)**	**Antidepressant classification**
Amoxapine	16	239	7.1 (4.3–11.7)	Tetracyclic antidepressant
Atomoxetine	1,079	17,353	6.6 (6.2–7.1)	Norepinephrine reuptake inhibitor
Maprotiline	18	302	6.3 (3.9–10.1)	Tetracyclic antidepressant
Mianserin	33	587	5.9 (4.2–8.4)	Tetracyclic antidepressant
Phenelzine	55	1,150	5.0 (3.9–6.6)	Monoamine oxidase inhibitor
Clomipramine	111	2,794	4.2 (3.5–5.1)	Tricyclic antidepressant
Fluvoxamine	116	2,985	4.1 (3.4–4.9)	Selective serotonin reuptake inhibitor
Olanzapine/Fluoxetine	95	2,647	3.8 (3.1–4.7)	Atypical antipsychotic/Selective serotonin reuptake inhibitor
Esketamine	12	337	3.8 (2.1–6.7)	Non-competitive N-methyl-D-aspartate receptor antagonist
Imipramine	82	2,401	3.6 (2.9–4.5)	Tricyclic antidepressant
Mirtazapine	827	24,504	3.6 (3.3–3.8)	Tetracyclic antidepressant
Doxepin	139	4,283	3.4 (2.9–4.1)	Tricyclic antidepressant
Escitalopram	1,175	39,569	3.2 (3.0–3.4)	Selective serotonin reuptake inhibitor
Desvenlafaxine	492	16,521	3.2 (2.9–3.5)	Serotonin-norepinephrine reuptake inhibitor
Nortriptyline	166	5,611	3.1 (2.7–3.6)	Tricyclic antidepressant
Paroxetine	1,537	53,007	3.1 (2.9–3.3)	Selective serotonin reuptake inhibitor
Venlafaxine	1,543	53,349	3.1 (2.9–3.3)	Serotonin-norepinephrine reuptake inhibitor
Citalopram	1,248	44,505	3.0 (2.8–3.2)	Selective serotonin reuptake inhibitor
Vilazodone	70	2,474	3.0 (2.4–3.8)	Serotonin modulator and stimulator
Duloxetine	1,710	61,438	3.0 (2.8–3.1)	Serotonin-norepinephrine reuptake inhibitor
Selegiline	40	1,521	2.8 (2.0–3.8)	Monoamine oxidase inhibitor
Trazodone	721	27,640	2.8 (2.6–3.0)	Serotonin antagonist and reuptake inhibitor
Amitriptyline	676	25,920	2.8 (2.6–3.0)	Tricyclic antidepressant
Tranylcypromine	15	583	2.7 (1.6–4.5)	Monoamine oxidase inhibitor
Fluoxetine	1,037	42,066	2.6 (2.5–2.8)	Selective serotonin reuptake inhibitor
Sertraline	1,638	67,904	2.6 (2.5–2.7)	Selective serotonin reuptake inhibitor
Bupropion	1,048	49,599	2.2 (2.1–2.4)	Norepinephrine-dopamine reuptake inhibitor
Milnacipran	58	2,858	2.1 (1.7–2.8)	Serotonin-norepinephrine reuptake inhibitor
Vortioxetine	52	4,075	1.3 (1.0–1.8)	Serotonin modulator and stimulator
Levomilnacipran	1	260	0.4 (0.1–2.9)	Serotonin-norepinephrine reuptake inhibitor

**Figure 2:**
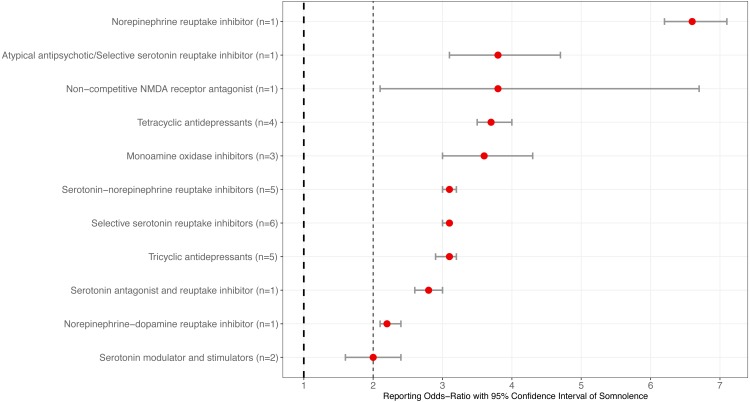
Association of somnolence based on antidepressant class from 30 compounds. Forest plots illustrating reporting odds-ratios (ROR) and 95% confidence intervals of antidepressant class ranked from the highest to lowest reporting odds of somnolence reported to the Food and Drug Administration Adverse Events Reporting System during January 2004 and September 2019. NMDA indicates N-methyl-D-aspartate.

When grouping the compounds based on pharmacological class, the selective serotonin reuptake inhibitor (SSRI) class of antidepressants had the most reported cases of somnolence (*n* = 6,751) ROR = 3.1 (95% CI [3.0–3.1]). Moreover, least number of somnolence cases was the non-competitive NMDA class and shown above for esketamine. The norepinephrine reuptake inhibitor atomoxetine, reported above, was associated with the highest reporting odds of somnolence. The serotonin modulator and stimulators (*n* = 122) ROR = 2.0 (95% CI [1.6–2.4]) had the lowest reporting odds of somnolence among the 11 tested classes of antidepressants. The following three classes of antidepressants were found to have equal reporting odds of somnolence: serotonin-norepinephrine reuptake inhibitors (SNRIs) (*n* = 3,804) ROR = 3.1 (95% CI [3.0–3.2]), SSRIs reported above, and tricyclic antidepressants (TCAs) (*n* = 1,174) ROR = 3.1 (95% CI [2.9–3.2]). Table 2 and Fig. 2 provide the final statistical findings and graphical illustration of the eleven antidepressant classes in this study.

**Table 2:** Reporting Odds-Ratios (ROR) and 95% confidence intervals of 30 antidepressant compounds based on antidepressant classification included in the study.

Antidepressant classification	**Somnolence cases**	**Other reported cases**	**Somnolence ROR (95% CI)**
Norepinephrine reuptake inhibitor (*n* = 1)	1,079	17,353	6.6 (6.2–7.1)
Atypical antipsychotic/Selective serotonin reuptake inhibitor (*n* = 1)	95	2,647	3.8 (3.1–4.7)
Non-competitive N-methyl-D-aspartate receptor antagonist (*n* = 1)	12	337	3.8 (2.1–6.7)
Tetracyclic antidepressants (*n* = 4)	894	25,632	3.7 (3.5–4.0)
Monoamine oxidase inhibitors (*n* = 3)	110	3,254	3.6 (3.0–4.3)
Serotonin-norepinephrine reuptake inhibitors (*n* = 5)	3,804	134,426	3.1 (3.0–3.2)
Selective serotonin reuptake inhibitors (*n* = 6)	6,751	250,036	3.1 (3.0–3.1)
Tricyclic antidepressants (*n* = 5)	1,174	41,009	3.1 (2.9–3.2)
Serotonin antagonist and reuptake inhibitor (*n* = 1)	721	27,640	2.8 (2.6–3.0)
Norepinephrine-dopamine reuptake inhibitor (*n* = 1)	1,048	49,599	2.2 (2.1–2.4)
Serotonin modulator and stimulators (*n* = 2)	122	6,549	2.0 (1.6–2.4)

## Discussion

### Somnolence and 5-HT2C, NET, and Alpha-1 adrenergic receptor binding affinities

This study assessed and ranked the reporting odds of somnolence among thirty antidepressant compounds using post-marketing adverse drug reactions reported to the FAERS. As shown in Table 1, seven groups of antidepressants resulted in equivalent reporting odds of somnolence. Consistent with the original hypothesis, the compound with the highest reporting odds of somnolence indeed has the strongest 5-HT2C receptor binding affinity, amoxapine (Ki = 2 nM), among the 30 antidepressant compounds tested (Roth et al., 2000; Tatsumi et al., 1997). Further, amoxepine’s binding affinities for NET and H1 receptor are Ki = 16 nM and 25 nM, respectively. Atomoxetine, a norepinephrine reuptake inhibitor and generally indicated in patients with ADHD, was ranked second in somnolence overall and binds to the NET with the lowest Ki value of 3.5 nM among the thirty antidepressant compounds in this study (Roth et al., 2000; Tatsumi et al., 1997). Mirtazapine, a tetracyclic antidepressant, was ranked 11th in reporting odds of somnolence and had the strongest H1 receptor binding affinity with a Ki of 0.14 nM (Roth et al., 2000; Tatsumi et al., 1997). When considering the glymphatic system, doxepin was the antidepressant compound with the lowest alpha-1 adrenergic receptor binding affinity (Ki = 23.4 nM) and ranked 12th in reporting odds of somnolence (*n* = 139) ROR = 3.4 (95% CI [2.9–4.1]), but also has a low H1 receptor Ki value of 0.24 nM (Roth et al., 2000; Tatsumi et al., 1997).

Maprotiline, a tetracyclic antidepressant, was ranked third among the thirty antidepressants and binds to the H1 receptor (Ki = 1.7 nM) and NET (Ki = 11.1 nM) with high affinity (Roth et al., 2000; Tatsumi et al., 1997). Similarly, mianserin, another tetracylic antidepressant, binds to the 5-HT2C and H1 receptors with Ki values of 2.59 nM and 1nM, respectively (Roth et al., 2000; Tatsumi et al., 1997). In contrast, vortioxetine, a serotonin modulator and stimulator, was least associated with somnolence and potently inhibits the serotonin transporter (SERT) with a Ki = 1.6 nM, however weakly binds to the 5-HT2C receptor with a Ki = 180 nM and the NET with a Ki = 113 nM (D’Agostino, English & Rey, 2015; Di & Kerns, 2015; Roth et al., 2000).

A recent meta-analysis that analyzed 276 studies and 14 second-generation antidepressants, evaluated somnolence in patients undergoing treatment for major depression, reported that fluvoxamine (NET = 1,892 nM, 5-HT2C Ki = 6,700 nM) and mirtazapine (H1 inverse agonist Ki=0.14 nM, 5-HT2C Ki = 39 nM) resulted in the highest odds of somnolence (Alberti et al., 2015). Moreover, in the same study, buproprion (NET Ki = 5,600 nM, H1 = 6,700 NM) was reported to have the lowest odds of somnolence and highest odds of insomnia (Alberti et al., 2015; Roth et al., 2000). Nevertheless, the odds of causing somnolence are not related to popularity index of antidepressants. A recent scientometric analysis reported that fluoxetine has the highest popularity index (4.0) (Tran et al., 2019). The popularity indexes of aforementioned antidepressants are as follows: fluvoxamine (1.00), bupropion (0.71), mirtazapine (0.61), vortioxetine (0.1), desvenlafaxine (0.08), vilazodone (0.04) and levomilnacipran (0.03). (Tran et al., 2019).

### Antidepressant-associated somnolence and CYP2C19 poor metabolizers

Patients who are CYP2C19 Poor Metabolizers—that is have single-nucleotide polymorphisms (SNPs) allele combinations of CYP2C19 *2/*2-3 and have a fraction of activity (FA) of 0.005—when compared to CYP219 Normal Metabolizers (*1/*1 FA = 1 and *2-3/*17 FA = 0.8) will have an increase in drug exposure (Fermier et al., 2018). The allele frequency of the functionally inactive CYP2C19*2 variant is seen at rates of 18.1% in African, 10.1% in Admixed American, 18.3% in European, 31.0% in East Asian, and 34.0% in Southeast Asian populations (Zhou, Ingelman-Sundberg & Lauschke, 2017). Based on the study results, the antidepressant compounds with the highest reporting odds of somnolence and not primarily metabolized by CYP2C19 substrates are: vilazodone, mirtazapine, and duloxetine. Further, antidepressants with the lowest reporting odds of somnolence and not primarily metabolized by CYP2C19 are: levomilnacipran, milnacipran, vortioxetine, and buproprion.

### Antidepressant-associated somnolence and CYP2D6 poor metabolizers

Increased drug exposures are also evident in patients who are prescribed CYP2D6 drug substrates and have CYP2D6 loss-of-function DNA sequence variants leading to being phenotypically classified as CYP2D6 Poor Metabolizers (*3-8/*3-8 FA = 0.01 and *10/*10 FA = 0.1) (Fermier et al., 2018). The CYP2D6*4 functionally inactive variant has the highest allele frequency rate and is seen in 11.9% in African, 15.7% in Admixed American, 15.5% in European, 0.4% in East Asian, and 11.6% in Southeast Asian populations (Zhou, Ingelman-Sundberg & Lauschke, 2017). The CYP2D6*5 inactive variant is seen at a 6.5% allele frequency in the Southeast Asian population (Zhou, Ingelman-Sundberg & Lauschke, 2017). Based on the study results, milnacipran and levomilnacipran are antidepressant compounds with low and no association with somnolence, respectively. Further, tablet-based antidepressants that are significantly associated with somnolence and have no to minor CYP2D6 contribution are: desvenlafaxine and mirtazapine. Esketamine is FDA approved in conjunction with an oral antidepressant (DailyMed., 2019b).

### Pharmacogenomics, therapeutic drug monitoring, and clinical pharmacologists

In cases where CYP450 metabolizer status are unknown or even unclear from pharmacogenomic testing reports due to concomitant medications resulting in drug-drug-gene interactions, directly measuring drug plasma concentrations via therapeutic drug monitoring (TDM) is often most efficient (Eugene & Eugene, 2018; Qin et al., 2019). Of note, CYP2C19 *1/*2-3 (FA = 0.3) genotypes are phenotypically CYP2C19 Intermediate Metabolizers and known to have a reduced fraction of activity relative to CYP2C19 Normal Metabolizers (FA = 1) (Fermier et al., 2018). In contrast, excessive metabolic rates, resulting in sub-therapeutic antidepressant plasma concentrations, are seen in patients who are CYP2C19 Ultra-rapid Metabolizers and have variant combinations of CYP2C19 *17/*17 (FA = 2.03) and *1/*17 (FA = 1.59) (Fermier et al., 2018). Thus, when considering the results for selecting an antidepressant with an association with somnolence, or even extrapolating from other studies, there may be wide inter-individual variability in antidepressant therapeutic effect and adverse effect. More importantly, the United States Food and Drug Administration expresses concern to physicians and other healthcare professionals that the association of current pharmacogenomics testing reports and antidepressant efficacy are not yet established (FDA, 2018).

In various countries, physicians specializing in clinical pharmacology routinely utilize pharmacogenomics as a means to avoid drug toxicity by mainly using TDM, applying Bayesian dosing methods, and other techniques with the aim of patient drug safety (Borobia et al., 2017; Eichelbaum, Dahl & Sjöqvist, 2018). In the U.S., there needs to be a significant increase in clinical pharmacology training programs that provide direct entry following medical school, as is done internationally, to have more clinical pharmacologists within the hospitals to carry out traditional patient care roles, consult with other physicians, interpret the pharmacogenomics testing reports, and advance the field. One major limitation of using pharmacogenomics testing reports *alone* to guide drug decision-making is that most patients are prescribed more than one medication resulting in drug-drug interactions as well as drug-drug-gene interactions. Whereas, adding TDM will increase precision in dosing and avoid toxicity based on established safety plasma ranges using pharmacokinetics (Hefner, 2018). Further, drug selection based on pharmacogenomics testing reports alone without also accounting for established efficacy is that often pharmacogenomic testing systematically recommends CYP3A4 substrate alone, as well as compounds not dependent on cytochrome P450 oxidative metabolism, despite established therapeutic efficacy for all compounds that gained regulatory approval. More specifically, a pharmacogenomics study found that among twenty-two antidepressants, desvenlafaxine and levomilnacipran (renally excreted) were recommended greater than 90% of the time, while in less than 10.5% of the time citalopram, duloxetine, escitalopram, fluoxetine, fluvoxamine, mirtazapine, paroxetine, and sertraline were never in the green (i.e., use as directed) category (Macaluso & Preskorn, 2018). However, in a systematic review and network meta-analysis comprised of 522 clinical trials with 116,477 participants found that (in order of decreased efficacy): amitriptyline, mirtazapine, duloxetine, venlafaxine, paroxetine, milnacipran, fluvoxamine, escitalopram, nefazodone, and sertraline were the top ten most efficacious; while levomilnacipran ranked 14th and desvenlafaxine was ranked 20th out of the 21 assessed antidepressants in the study (Cipriani et al., 2018).

### Study limitations

The study limitations are that the reporting odds-ratios are generally hypothesis generating due to the entire population of patients experiencing the side-effect are not known, but are dependent on voluntary submission of the adverse event to the FDA. Further, the term somnolence may be reported and recorded as sedation and this study did not factor in the term sedation in the analysis. For example, in the adverse reactions section of atomoxetine’s package insert states that both somnolence and sedation was recorded as somnolence (DailyMed., 2018). For the newest compound in this study, the FAERS results for esketamine showed 61 adverse events for sedation, 288 as other cases, and 349 total reported cases resulting in a ROR = 140 (95% CI [111–176]) for sedation alone. Statistically, the overall interpretation of the results are that the strength of the ADR association with the drugs-under-test and are not indicative of risk (Montastruc et al., 2011). Further, psychotropic medications exhibit target and off-target effects influencing the cholinergic (acetylcholine), gamma-aminobutyric acid (GABA)-ergic, glutaminergic (glutamate), glycinergic, and L-Arginine nitric oxide pathways, among others, so the association of somnolence may not be directly associated with the glymphatic system alone.

## Conclusions

The top three antidepressant compounds with the strongest reporting odds of somnolence are amoxapine, atomoxetine, and maprotiline. Levomilnacipran was not associated with somnolence. Vortioxetine, milnacipran, and bupropion were least associated with somnolence. Consistent with the original hypothesis, amoxepine has strongest 5-HT2C receptor binding affinity and has the highest reporting odds of somnolence. Atomoxetine, ranked second in reporting odds of somnolence, binds to the NET with the strongest binding affinity. Lastly, mirtazapine has the strongest H1 receptor binding affinity among the thirty antidepressant compounds and was ranked 11th in reporting odds of somnolence. This study provides an informative ranking of reporting odds of somnolence among thirty antidepressant compounds with an already wide array of clinical indications and provides insight to potential drug repurposing in psychopharmacology.

## Additional Information and Declarations

### Data Availability

The following information was supplied regarding data availability: Cases reported in this article are freely available from the United States Food and Drug Administration and may be downloaded here: https://www.fda.gov/drugs/fda-adverse-event-reporting-system-faers/fda-adverse-event-reporting-system-faers-latest-quarterly-data-files.
